# Nanoceria-mediated delivery of doxorubicin enhances the anti-tumour efficiency in ovarian cancer cells via apoptosis

**DOI:** 10.1038/s41598-017-09876-w

**Published:** 2017-08-25

**Authors:** Joydeep Das, Yun-Jung Choi, Jae Woong Han, Abu Musa Md Talimur Reza, Jin-Hoi Kim

**Affiliations:** Department of Stem Cell and Regenerative Biotechnology, Humanized Pig Research Center (SRC), Konkuk University, Seoul, 143-701 South Korea

## Abstract

Nanocarriers are widely used for effective delivery of anticancer drugs to tumours with potential to improve cancer treatment. Here, we developed a nanoceria (CeO_2_)-based system for delivery of the anti-cancer drug doxorubicin (DOX) to human ovarian cancer cells. Negatively charged nanoceria could conjugate with the cationic DOX via electrostatic interaction under physiological conditions, forming DOX-loaded nanoceria (CeO_2_/DOX). CeO_2_/DOX particles displayed nearly spherical shapes, along with superior drug-loading content (22.41%), loading efficiency (99.51%), and higher cellular uptake and drug release behaviours compared to free DOX. Moreover, DOX was released faster from CeO_2_/DOX under reductive acidic conditions (pH 5.0, 10 mM glutathione) than under physiological conditions (pH 7.4). The initial intracellular DOX concentration was higher in the free DOX groups than in the CeO_2_/DOX groups, but quickly reduced to 25% of the initial concentration after 24-h culture. By contrast, CeO_2_/DOX showed sustained DOX release over time and maintained a high intracellular DOX concentration for up to 72 h. *In vitro* assays showed that CeO_2_/DOX exhibited higher cell proliferation inhibition and apoptosis compared with free DOX. These results highlight DOX-loaded nanoceria as a promising therapeutic agent for cancer treatment.

## Introduction

Ovarian cancer is the fifth most prevalent cancer among women causing death and is the most lethal gynaecologic malignancy, mainly owing to late-stage diagnosis. If the cancer is detected in its earliest stages, more than 90% of the patients have a better prognosis. In the last few decades, new treatment modalities with improved diagnostic methods and surgical techniques were established, but only a marginal survival improvement was gained^1^. Most patients will ultimately recur and succumb to their disease. In many cases, chemotherapy helps to improve the overall survival of patients with ovarian cancer^2^. Many chemotherapeutic drugs are currently used in clinical practice, such as doxorubicin (DOX), cisplatin, decitabine, paclitaxel, gemcitabine, cyclophosphamide, carboplatin, and their combinations, for ovarian cancer treatment^3^. However, there is an urgent need to identify new therapeutic agents that can improve the efficacy of existing therapeutic modalities.

Nanotechnology is a rapidly growing field towards the development of nanomedical products to improve therapeutic strategies against cancer, and have been shown to improve the pharmacodynamic and pharmacokinetic properties of conventional chemotherapeutic agents and enhance their efficacy with less toxicity^4^. Nanoceria, or cerium oxide (CeO_2_), is a rare-earth metal oxide with the unique ability to switch between Ce^4+^ and Ce^3+^ depending on the environment^5^. Karakoti *et al*.^6^ reported that nanoceria shows active switching from Ce^4+^ to Ce^3+^ in acidic medium, whereas the higher oxidation state is more stabilized in basic medium. Nanoceria possesses excellent antioxidant properties because of its ability to switch between mixed oxidation states^7, 8^. Walkey *et al*.^9^ demonstrated that nanoceria is emerging as an antiinflammatory material. They also reported that the interaction with biological molecules such as proteins, lipids and anions alter the behaviour of nanoceria *in vivo*. Nanoceria can interact with phosphate ester bonds of biologically relevant molecules, and the dephosphorylation reaction depends on the availibility of Ce(III) sites and is inhibited when Ce(III) is converted to Ce(IV)^10^. However, nanoceria do not dephosphorylate DNA^10, 11^. Nanoceria shows a pH-dependent effect on reactive oxygen species (ROS) generation^12^. It has been reported that nanoceria acts primarily as a scavenger of H_2_O_2_ in (neutral) normal tissues but as a producer of H_2_O_2_ in an (acidic) cancer environment^13^. The pro and anti-oxidant properties of nanoceria depent on several other factors, such as, particle shape and size, surface chemistry, and surface additives or ligands that can participate in redox reactions^14, 15^. However, the toxicity and cellular interaction are largely dependent on the physicochemical properties of nanoceria^16^. Nanoceria has been shown to produce a sustained regression of oxidative stress-induced neovascularizations, prevent pathologic vascular leakage and inhibit in young adult vldlr(-/) mice^17^. Besides, the use of nanoceria as a potential drug delivery system has also been reported by several researchers^18, 19^.

Giri *et al*.^20^ showed that nanoceria could act as a therapeutic agent in ovarian cancer cells both *in vitro* and *in vivo*. Specifically, they showed that nanoceria (containing 63% Ce^3+^) could inhibit ROS generation and vascular endothelial growth factor-induced proliferation, and could attenuate cell migration and invasion without affecting cell proliferation. Furthermore, the same group^21^ later showed that folic acid-conjugated nanoceria (FA-CeO_2_ containing 24% Ce^3+^) inhibited ovarian cancer cell proliferation (due to increased cellular uptake) and increased ROS production. Despite these differences in its pro- or anti-oxidant nature, both studies demonstrated an association of nanoceria with significant reduction in tumour growth and attenuation of angiogenesis in an ovarian cancer nude mouse model. Moreover, they also showed that the combination of FA-CeO_2_ with cisplatin decreased the tumour burden significantly, even compared to the cisplatin alone-treated group. Similar kind of anti-invasive effects of nanoceria on human melanoma cells as well as antitumor and antiangiogenic effects in *in vivo* tumor model were observed^22^. Sack *et al*.^23^ showed a synergistic effect on cytotoxicity and oxidative damage after co-incubation with nanoceria and DOX in human melanoma cells, whereas nanoceria protected human dermal fibroblasts against DOX-induced cytotoxicity. Nanoceria has also been shown to have anticancer activity in several other types of cancers, including in colon, cutaneous squamous, and pancreatic cancer models^12, 13, 24^, and the increased generation of ROS was believed to be one of the mechanisms contributing to its anti-tumour effect.

DOX is one of the most widely used chemotherapeutic drugs for the treatment of various kinds of cancers, including ovarian cancer^3, 25, 26^. However, its efficiency is limited owing to the short intracellular retention with lower applicable dosages. The clinical use of DOX is also restricted due to its profound cardiotoxicity, which can cause irreversible cardiomyopathy and/or heart failure^27, 28^. In the current study, we conjugated DOX with nanoceria to enhance its cytotoxicity, and investigated the potential of CeO_2_/DOX nanocomplexes in inhibiting ovarian cancer growth *in vitro*. Herein, we propose that high anticancer efficiency can be achieved by combining the advantages of DOX as a conventional chemotherapeutic drug and the anti-tumorigenic nature of nanoceria to prepare a one-particle system (CeO_2_/DOX).

## Results

### Preparation and characterization of CeO_2_ and CeO_2_/DOX complexes

**Figure 1:**
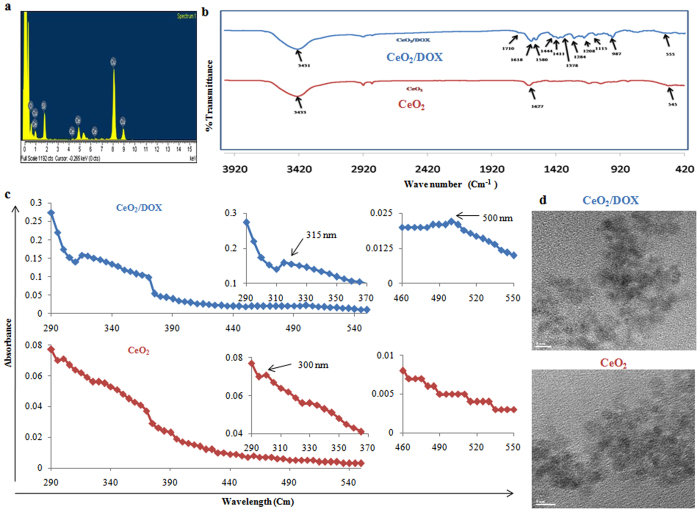
Characterization of CeO_2_ and CeO_2_/DOX nanoparticles. (**a**) EDS spectrum of CeO_2_; (**b**) FTIR spectrum of CeO_2_ and CeO_2_/DOX; (**c**) UV-VIS spectra of CeO_2_ and CeO_2_/DOX; (**d**) TEM images of CeO_2_ and CeO_2_/DOX.

Nanoceria (CeO_2_) was prepared according to the method described in our previous publication^11^ by simply refluxing ammonium cerium (IV) nitrate and urea. Characterization of the synthesized CeO_2_ was achieved by EDS and FTIR spectroscopic analyses. The EDS spectrum depicted characteristic peaks of Ce and O (Fig. 1a). In addition, a Cu peak arising from the TEM grid and an Si peak from the detector were observed. The chemical nature of CeO_2_ was also verified from the FTIR spectrum, which showed a characteristic absorption band at 500 to 550 cm^−1^ due to the Ce-O stretching vibration (Fig. 1b). Infrared absorption bands were also observed at 3433 cm^−1^ and 1627 cm^−1^ due to water molecules adsorbed on the nanoparticle surface (Fig. 1b). We have also checked the crystal structure (by XRD analysis) of the synthesized CeO_2_ nanoparticles. Figure S1a shows the X-ray diffraction (XRD) pattern of the synthesized nanoceria. The high intensity peaks were observed at 29.08, 32.98, 47.72, 56.94, 69.84 and 77.78 respective to the (111), (200), (220), (311), (400) and (331) crystal planes. These diffraction peaks indicates a cubic fluorite structure^18, 29, 30^. The nanoceria were further examined by X-ray photoelectron spectroscopy (XPS) to determine the different valence states. Figure S1b shows a relatively higher abundance of Ce^4+^ corresponding to the binding energy peaks at 883, 889, 899, 902, 908, and 917 eV^18, 31, 32^. The indicative peaks of Ce^3+^ at 880, 885 and 900 eV are suppressed by high intensity Ce^4+^ peaks^18, 31, 32^. However, a clear indicative peak of Ce^3+^ is present at 905 eV position. Therefore, both the +3 and +4 oxidation states are present in nanoceria, but the major valence of Ce is 4+. McCormack *et al*.^33^ reported that nanoceria with higher percentage of surface Ce^+3^ oxidation states are more prone to interact with phosphate ions and forming cerium phosphate, thereby influencing the catalytic activities of nanoceria. As we have seen that our synthesized nanoceria showed higher abundance of Ce^4+^ oxidation states (confirmed by XPS data), we expect less interaction with phosphate ions. Therefore, we can practically ignore the effects of phosphate ions on the physiological functions of nanoceria and used PBS to suspend CeO_2_ and CeO_2_/DOX nanocomplex.

After confirming the synthesis of CeO_2_, DOX was loaded onto CeO_2_ via electrostatic interactions. The amount of DOX bound to the nanoceria surface was determined by UV absorption measurements at 480 nm. The DLE and DLC contents were 99.51% and 22.41%, respectively (Table 1). The adsorption of DOX on the CeO_2_ surface was also confirmed by FTIR analysis. The FTIR spectrum of DOX-loaded CeO_2_ showed multiple characteristic peaks of DOX (Fig. 1b). The broad peak at 3431 cm^−1^ can be assigned to the stretching band of -OH groups. The peak at 1710 cm^−1^ is due to the stretching band of C=O groups; peaks at 1618 cm^−1^ and 1580 cm^−1^ are due to the bending band of N-H groups; the peak at 1411 cm^−1^ is due to C-C stretching; the peak appearing at 1284 cm^−1^ is due to framework vibration of the carbonyl group in DOX’s anthracene cycle; the peak at 1208 cm^−1^ is due to C-O-C asymmetric stretching vibration; and the peak at 987 cm^−1^ is due to C-O stretching of the alcohol group^34–37^. These results indicate that DOX was successfully loaded onto the CeO_2_ nanoparticles.

**Table 1:** Drug loading content (DLC) and drug loading efficiency (DLE) of CeO_2_/DOX nanoparticles.

Feed ratio, CeO_2_: DOX (w/w)	DLC %	DLE %
5: 1.45	22.41	99.51

The optical properties of synthesized CeO_2_ were checked by acquisition of the UV spectrum, which showed a distinct absorption peak at 300 nm and was devoid of impurity peaks (Fig. 1c). However, in the case of CeO_2_/DOX, the absorption peak appeared at 315 nm (Fig. 1c), which indicated a larger particle size. The CeO_2_/DOX complex also showed a characteristic peak of loaded DOX at 500 nm (Fig. 1c), which was absent in the spectrum of CeO_2_ nanoparticles. TEM analysis showed that the synthesized CeO_2_ particles were almost spherical, with diameters in the range of 3–4 nm (Fig. 1d). However, the apparent increase in the size of the CeO_2_/DOX complexes was not clear from the TEM images.

The processes of DOX deposition on the CeO_2_ surface and complex formation between CeO_2_ and DOX were also monitored by DLS and zeta-potential analyses. The DLS data suggested that the average diameter of CeO_2_ and CeO_2_/DOX particles was 208 ± 9 nm and 308 ± 5 nm, respectively (Fig. S2). The size of the CeO_2_ particles increased by approximately 100 nm when loaded with DOX. The size of nanoparticles appear bigger by DLS compared to by TEM analysis due to extensive solvation/hydration of nanoparticles. The results can be explained by the fact that by DLS measurement, the average diameter is calculated from the diffusional properties of dynamic nanoparticles in hydrated state. On the other hand, by TEM analysis, the average primary particle diameter is calculated in static and dried state^38, 39^. The polydispersity index of the synthesized CeO_2_ and CeO_2_/DOX complexes was below 0.3, indicating a uniform and homogeneous size distribution. The zeta potential of CeO_2_ was −27 ± 1.6 mV in PBS (pH 7.4), which increased to −16 ± 0.6 mV after DOX loading due to the association with positively charged DOX (Fig. S2).

### In vitro DOX release from CeO_2_/DOX complexes

**Figure 2:**
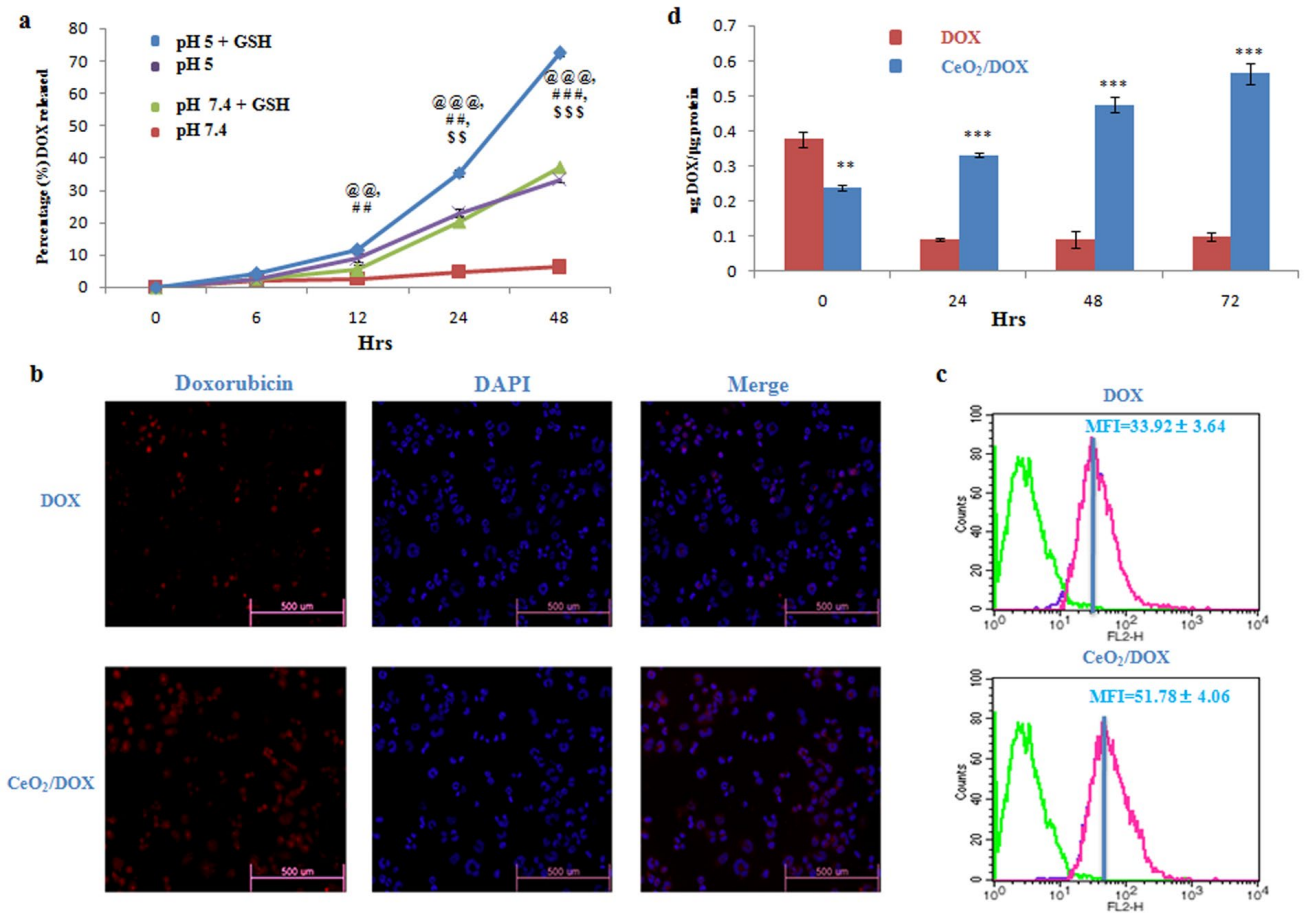
Intracellular uptake of CeO_2_/DOX nanoparticles and release of DOX from CeO_2_/DOX nanoparticles. (**a**) DOX release profiles of the CeO_2_/DOX nanoparticles in PBS under different conditions at 37 °C. The GSH concentration was fixed at 10 mM. The equivalent DOX concentration was 5 μg/mL. ^@^p < 0.05, ^@@^p < 0.01 and ^@@@^p < 0.01 versus the pH 7.4 group, ^#^p < 0.05, ^##^p < 0.01 and ^###^p < 0.01 versus the pH 7.4,GSH group, ^$^p < 0.05, ^$$^p < 0.01 and ^$$$^p < 0.01 versus the pH 5 group. (**b,c**) Cellular uptake of free DOX and CeO_2_/DOX nanoparticles after incubation of A2780 cells with a 2 μg/mL equivalent DOX concentration for 3 h, measured by fluorescence microscopy and FACS; MFI, mean fluorescence intensity. (**d**) Quantitative evaluation of intracellular DOX released from CeO_2_/DOX. A2780 cells were first treated with a 2 μg/mL equivalent DOX concentration for 3 h (taken as the 0 time-point), washed, and left untreated for a further 24, 48, and 72 h in DOX-free medium. All values are expressed as mean ± SD. *p < 0.05, **p < 0.01, and **p < 0.001 versus the free DOX-treated group.

The time-dependent *in vitro* release of DOX from CeO_2_/DOX complexes was investigated under physiological conditions (PBS, pH 7.4) and in a mildly acidic environment (pH 5.0) simulating the endo-lysosomal pH, as well as in combination with GSH (10 mM) that is present in high concentrations within lysosomes. In neutral PBS (pH 7.4), only a very small amount of DOX was released from CeO_2_/DOX in a very slow fashion, and the cumulative release of DOX was only about 6.23% within 48 h (Fig. 2a). In PBS of pH 5.0, the release rate of DOX from CeO_2_/DOX became much faster. The cumulative release of DOX from CeO_2_/DOX could reach as high as about 33.37% within 48 h, which was approximately 5.4-times higher than that observed at pH 7.4 (Fig. 2a). This result demonstrated that the release of DOX from CeO_2_/DOX nanoparticles was pH-sensitive. However, we have also checked the release profile of DOX from CeO_2_/DOX nanoparticles in medium mimicking the *in vivo* environment, such as PBS (pH = 7.4) containing 10% serum and observer that the cumulative release of DOX was only about 6% within 48 h (Fig. S1c).

It is noteworthy that the GSH addition to the release medium had a significant influence on the release rates of DOX from the nanocomplexes. The percentage of released DOX (72.35%) within the first 48 h under reductive conditions (pH 5.0, GSH 10 mM) was much higher than that (33.37%) observed at pH 5.0 (Fig. 2a). However, only 35.45% and 22.78% of the DOX was released within the first 24 h under the reductive condition (pH 5.0, GSH 10 mM) and at pH 5.0, respectively, indicating that the drug–nanoparticle interaction is very strong, so that DOX is released in a slow manner (Fig. 2a).

### Intracellular uptake of CeO_2_/DOX nanoparticles

The endocytosis of free DOX and DOX-loaded nanoparticles was compared in A2780 human ovarian cancer cells by both fluorescence microscopy and flow cytometry analysis. Since DOX itself is fluorescent, no additional markers were used. The fluorescence intensity is proportional to the amount of DOX internalized by the cells. Fluorescence microscopic images are shown in Fig. 2b. For both free DOX and CeO_2_/DOX nanoparticles, cellular uptake was observed after 3 h of incubation. DOX accumulation in both the cytoplasm and the nucleus was higher for CeO_2_/DOX than for free DOX.

The mechanism of cellular uptake of CeO_2_/DOX complexes into A2780 cells was investigated by pre-treating the cells with several endocytosis inhibitors prior to treatment with CeO_2_/DOX. The cellular uptake of the CeO_2_/DOX complexes was then qualitatively assessed by fluorescence microscopic analysis. Cells were pre-treated with several endocytosis inhibitors, including 5 μg/mL CPZ (inhibitor of clathrin-mediated endocytosis), 1 mM MBCD (suppressor or inhibitor of caveolae-mediated endocytosis), and 65 μM LY294002 (inhibitor of macropinocytosis). Pre-treatment with CPZ did not influence the fluorescence intensity of cells incubated with CeO_2_/DOX (Fig. S3). In contrast, pre-treatment with MBCD and LY294002 reduced the fluorescence intensity of cells incubated with CeO_2_/DOX compared to that of non-treated cells (Fig. S3). This result demonstrated that the CeO_2_/DOX complexes were selectively taken up by A2780 cells through both caveolin-mediated endocytosis and macropinocytosis.

The cellular uptake of free DOX and CeO_2_/DOX was further quantitatively checked by flow cytometry analysis. The mean fluorescence intensity was taken to quantitatively compare the endocytosis of DOX between groups. CeO_2_/DOX showed a 1.5-fold higher fluorescence intensity than free DOX (Fig. 2c). Collectively, these results indicate that CeO_2_/DOX shows higher cellular uptake via an endocytosis process than free DOX.

### Intracellular DOX release and retention

According to the *in vitro* DOX release profile of CeO_2_/DOX nanoparticles (Fig. 2a), it could be expected that CeO_2_/DOX would also show a time-dependent drug release pattern within the cells. A2780 cells were first treated with a 2 μg/mL equivalent DOX concentration for 3 h (taken as the 0 time-point), and the medium was changed. The intracellular DOX concentration was measured at 24-, 48-, and 72-h intervals. The intracellular DOX concentration was higher for the free DOX (0.38 ng/μg protein) treatment groups than in the CeO_2_/DOX (0.24 ng/μg protein) treatment groups after 3 h of treatment (at the 0 time-point). However, in the free DOX treatment groups, the intracellular DOX concentration quickly decayed (0.09 ng/μg protein) to 25% of the original concentration at the 24-h mark, and stayed at a similar level until 72 h (Fig. 2d). By contrast, the CeO_2_/DOX groups showed sustained release of DOX over time and maintained a high intracellular DOX concentration up to 72 h (Fig. 2d).

### Anti-tumour activity and apoptosis of CeO_2_ and CeO_2_/DOX

**Figure 3:**
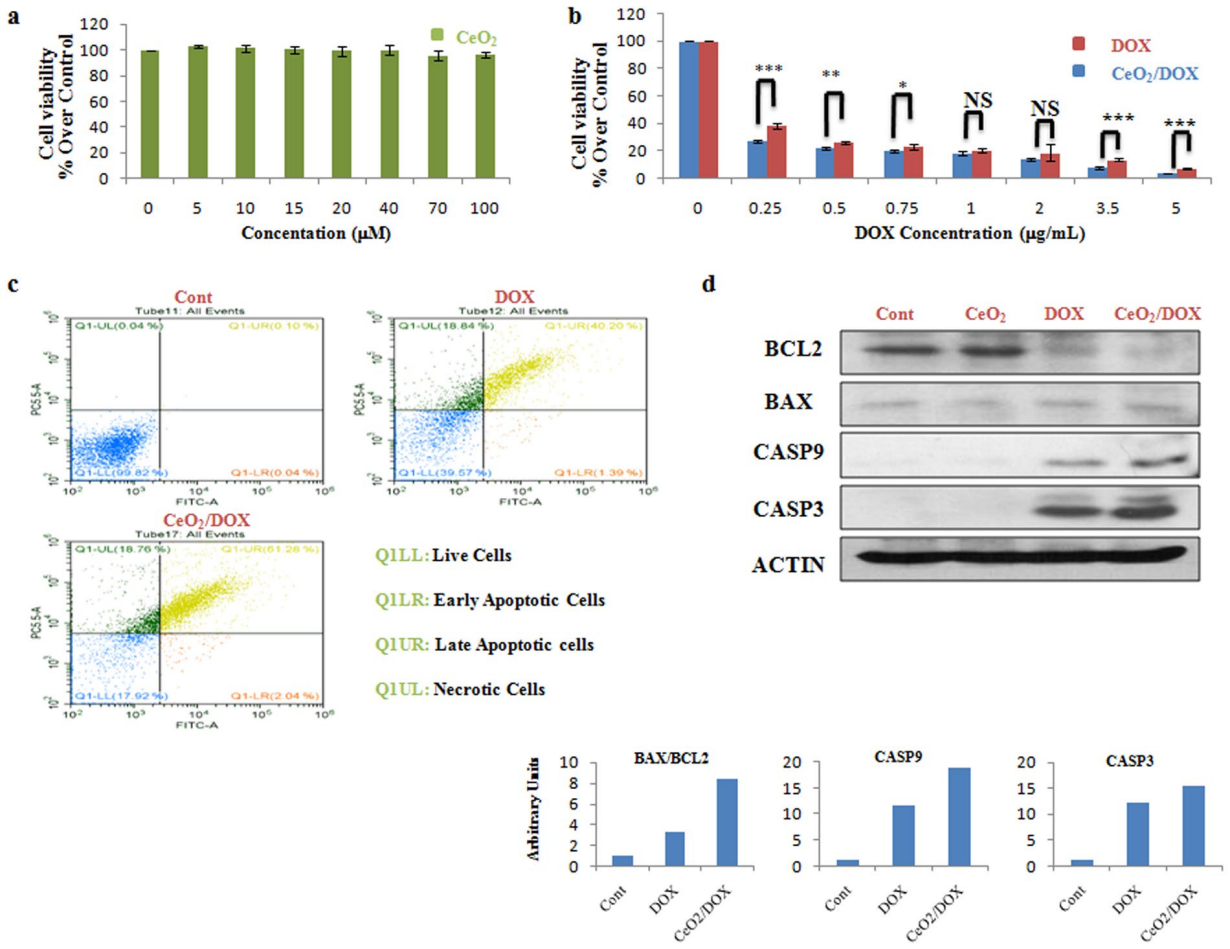
*In vitro* cytotoxicity of CeO_2_/DOX in A2780 ovarian cancer cells. (**a,b**) Cell viability relative to the control (100%). Cells were treated with different concentrations of CeO_2_, free DOX, or CeO_2_/DOX for 3 h. The cells were then washed and further cultured in fresh media for 72 h in the absence of any DOX and nanoparticles. All values are expressed as mean ± SD. *p < 0.05, **p < 0.01, and ***p < 0.001 versus the free DOX-treated group. (**c**) Apoptosis (dot plot distribution and % apoptosis calculations) measured by Annexin V/PI assay after exposure to 0.25 μg/mL equivalent doxorubicin for 3 h followed by washing and cultured for further 72 h in DOX free media. (**d**) Western blot analysis of apoptotic proteins. Cells were treated with a 0.25 μg/mL equivalent DOX concentration (either free DOX or CeO_2_/DOX) or with 5 μM CeO_2_ for 3 h, washed, and further cultured for 72 h. Densitometric analysis was carried out using Image J software.

The *in vitro* cytotoxicity of CeO_2_ nanoparticles was evaluated using a WST-8 assay. Three different human ovarian cancer cell lines, A2780, SKOV-3, and CAOV-3, were used for this assay. As shown in Fig. 3a, the viability of the A2780 cells treated with CeO_2_ was 96–100% at all test concentrations from 5 to 100 μM, revealing no significant effect on cell proliferation or survival. The *in vitro* anti-tumour activity of DOX-loaded CeO_2_ nanoparticles was also evaluated in the A2780, SKOV-3, and CAOV-3 cells. The cells were incubated with various concentrations of DOX for 3 h and then the medium was replaced with drug-free medium and culture continued for a further 72 h. The cell viabilities were evaluated thereafter and compared between the free-DOX and CeO_2_/DOX treatment groups. As shown in Fig. 3b, CeO_2_/DOX exhibited dose-dependent cell proliferation inhibition for A2780 cells. The results also showed that CeO_2_/DOX appeared to induce a higher anti-tumour effect compared with free DOX for all test concentrations (Fig. 3b). The difference in free DOX- and CeO_2_/DOX-induced cell death was further supported by AO/EB double-staining analysis at a test concentration of 0.25 μg/mL. AO can enter both live and apoptotic cells where it emits green fluorescence, whereas EB can only enter apoptotic cells where it emits red fluorescence^40^. The AO/EB assay showed that the percentage of apoptotic cells significantly increased following free DOX or CeO_2_/DOX exposure (Fig. S4a). The quantitative apoptotic activities of free DOX and CeO_2_/DOX on A2780 cells were further evaluated by flow cytometry. Cells have been double stained for viability (negative for PI) and apoptosis (positive for Annexin V-FITC). Free DOX and CeO_2_/DOX resulted in 41.6 and 63.32% apoptotic cells respectively, whereas the necrotic cell population did not differ (19%) among the groups (Fig. 3c).

To explore the possible signalling pathways through which DOX-loaded nanoparticles induced greater anticancer activity, we further evaluated the changes in the expression levels of apoptosis-related proteins by western blot analysis at the lowest test concentration applied (0.25 μg/mL). We observed that the expression of the apoptosis-regulating protein BCL-2 was down-regulated in both the free-DOX and CeO_2_/DOX groups, whereas BAX expression was not substantially altered when compared with that of the control group (Fig. 3d). However, the ratio of BAX to BCL-2 protein expression was more obviously increased in the CeO_2_/DOX group (8.33-fold) than in the free-DOX group (3.18-fold). As shown in Fig. 3d, the expression of cleaved caspase-9 was up-regulated in both the free-DOX and CeO_2_/DOX groups compared with the control group (Fig. 3d), and the increase was much more dramatic in the CeO_2_/DOX group (18.83-fold) than in the free-DOX group (11.68-fold). A similar result was detected for cleaved caspase-3 (Fig. 3d). Notably, the protein expression level in the CeO_2_-only group did not obviously differ compared with that of the control group (Fig. 3d).

**Figure 4:**
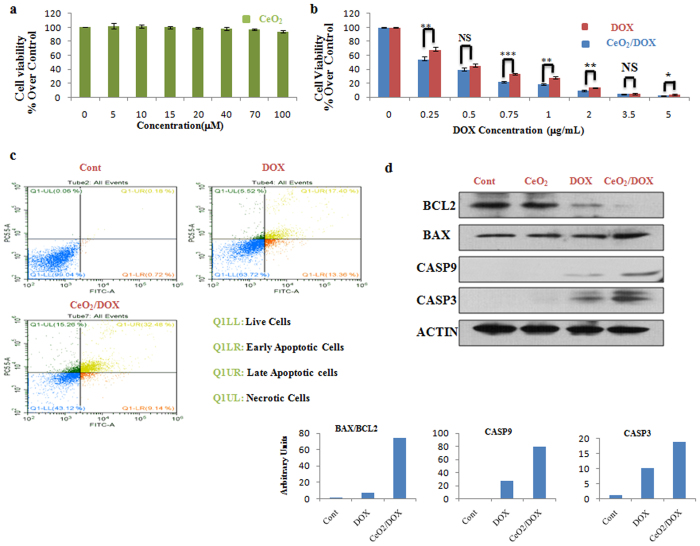
*In vitro* cytotoxicity of CeO_2_/DOX in CAOV3 ovarian cancer cells. (**a,b**) Cell viability relative to the control (100%). Cells were treated with different concentrations of CeO_2_, free DOX, or CeO_2_/DOX for 3 h. The cells were then washed and further cultured in fresh media for 72 h in the absence of any DOX and nanoparticles. All values are expressed as mean ± SD. *p < 0.05, **p < 0.01, and ***p < 0.001 versus the free DOX-treated group. (**c**) Apoptosis (dot plot distribution and % apoptosis calculations) measured by Annexin V/PI assay after exposure to 0.25 μg/mL equivalent doxorubicin for 3 h followed by washing and cultured for further 72 h in DOX free media. (**d**) Western blot analysis of apoptotic proteins. Cells were treated with a 0.25 μg/mL equivalent DOX concentration (either free DOX or CeO_2_/DOX) or with 5 μM CeO_2_ for 3 h, washed, and further cultured for 72 h. Densitometric analysis was carried out using Image J software.

Similar to A2780 cells, CeO_2_ also showed no significant effect on cell proliferation or survival in CAOV3 cells (Fig. 4a). Figure 4b shows that CeO_2_/DOX exhibited dose-dependent cell proliferation inhibition for CAOV3 cells, and appeared to induce a higher *in vitro* anti-tumour effect compared with free DOX at all test concentrations. The occurrence of apoptosis was also supported by AO/EB dual staining and AnnexinV/PI assay following free DOX or CeO_2_/DOX exposure at 0.25 μg/mL test concentration (Figs 4c, S4b). Free DOX and CeO_2_/DOX resulted in 30.8 and 41.6% apoptotic cells and 5.5 and 15.3% necrotic cells respectively (Fig. 4c). Immunoblotting analysis (Fig. 4d) further revealed that CeO_2_/DOX significantly increased the ratio of BAX to BCL-2 protein expression (74.4-fold), cleaved caspase-9 (80-fold), and caspase-3 (18.66-fold) compared with those of the free DOX group (6.4-fold, 28-fold, and 10.1-fold, respectively) at the lowest applied concentration (0.25 μg/mL).

**Figure 5:**
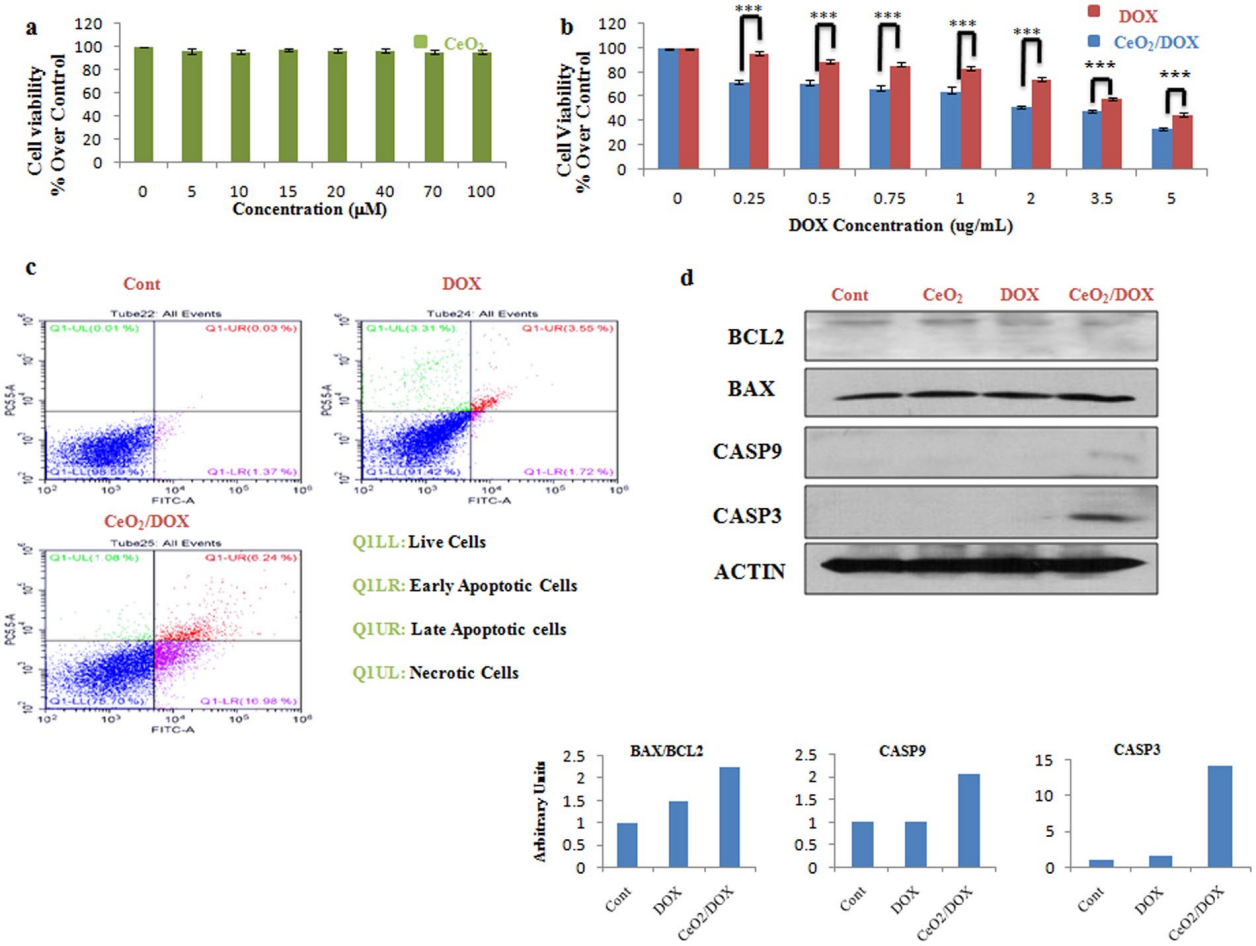
*In vitro* cytotoxicity of CeO_2_/DOX in SKOV3 ovarian cancer cells. (**a,b**) Cell viability relative to the control (100%). Cells were treated with different concentrations of CeO_2_, free DOX, or CeO_2_/DOX for 3 h. The cells were then washed and further cultured in fresh media for 72 h in the absence of any DOX and nanoparticles. All values are expressed as mean ± SD. *p < 0.05, **p < 0.01, and ***p < 0.001 versus the free DOX-treated group. (**c**) Apoptosis (dot plot distribution and % apoptosis calculations) measured by Annexin V/PI assay after exposure to 0.25 μg/mL equivalent doxorubicin for 3 h followed by washing and cultured for further 72 h in DOX free media. (**d**) Western blot analysis of apoptotic proteins. Cells were treated with a 0.25 μg/mL equivalent DOX concentration (either free DOX or CeO_2_/DOX) or with 5 μM CeO_2_ for 3 h, washed, and further cultured for 72 h. Densitometric analysis was carried out using Image J software.

Finally, we evaluated the cytotoxicity of the CeO_2_ nanoparticles in SKOV3 cells and compared the *in vitro* anti-tumour effect between free DOX and CeO_2_/DOX nanoparticles. CeO_2_ did not induce any significant cell proliferation inhibition (Fig. 5a), and CeO_2_/DOX exhibited a higher *in vitro* anti-tumour effect than free DOX within the concentration range of 0.25–5 μg/mL (Fig. 5b). However, at the concentration of 0.25 μg/mL, free DOX did not induce significant apoptotic cell death as evident from AO/EB dual staining, AnnexinV/PI assay and immunoblotting analysis (Figs 5c,d, S4c). On the other hand, CeO_2_/DOX induced extensive apoptosis as evident from AO/EB dual staining, AnnexinV/PI assay and increased expression of cleaved Caspase-9 (2 fold) and Caspase-3 (14.29 fold) (Figs 5c,d, S4c). Similar to the cell viability assay, the tendency towards increased apoptotic activity of CeO_2_/DOX in all tested human ovarian cancer cell lines is probably due to its ability for higher cellular uptake through endocytosis and sustained DOX release.

## Discussion

**Figure 6:**
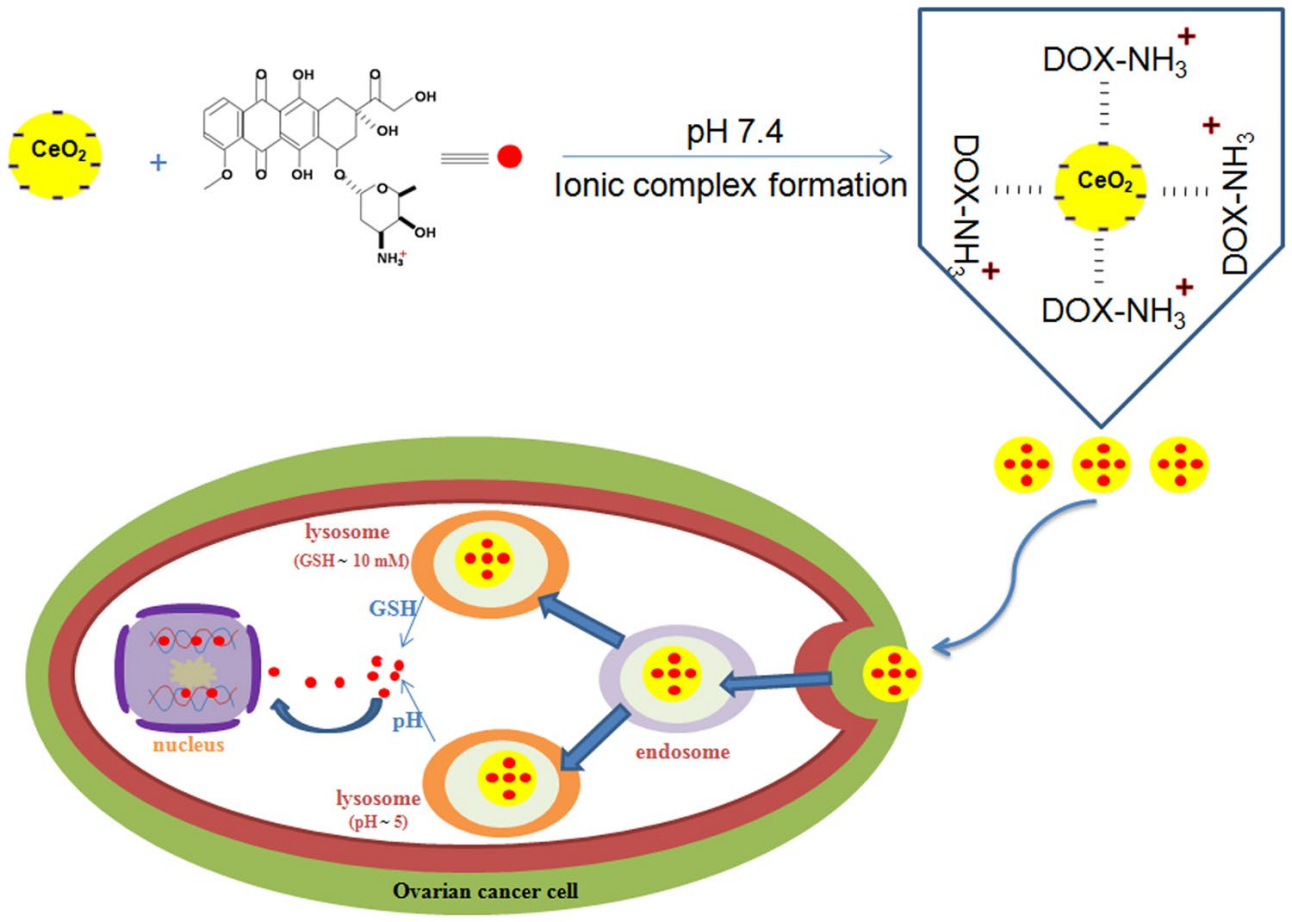
Schematic diagram of CeO_2_/DOX nanoparticle preparation, uptake in ovarian cancer cells and release of DOX.

In the present study, we have developed a new nanoceria (CeO_2_)-based drug delivery system wherein a model cationic anticancer drug, DOX, was loaded onto negatively charged CeO_2_ nanoparticles via simple electrostatic interaction for *in vitro* drug delivery applications to human ovarian cancer cells (Fig. 6).

Nanoceria (CeO_2_) was prepared by simply refluxing ammonium cerium (IV) nitrate and urea as described in our previous publication^11^. After confirming the synthesis of CeO_2_ by EDS and FTIR spectroscopic analyses, DOX was loaded onto CeO_2_ via electrostatic interactions. The adsorption of DOX on the CeO_2_ surface was confirmed by multiple characteristic peaks of DOX obtained from the FTIR spectrum of DOX-loaded CeO_2_. The DLE and DLC contents were 99.51% and 23.37%, respectively. The processes of DOX deposition on the CeO_2_ surface and complex formation between CeO_2_ and DOX were also monitored by UV-VIS spectroscopy and zeta-potential analyses. The CeO_2_/DOX complex displayed a characteristic peak of loaded DOX at 500 nm. The zeta potential of CeO_2_ was increased by approximately 11 mV when loaded with DOX due to the association with positively charged DOX.

This synthesized CeO_2_/DOX could maintain strong drug–nanoparticle electrostatic interactions under physiological conditions, and exhibited pH/reduction dual-responsive drug release behaviour. However, CeO_2_/DOX nanoparticles are also stable in medium mimicking the *in vivo* environment, such as PBS (pH = 7.4) containing 10% serum. Therefore, we can conclude that the CeO_2_/DOX system is stable in *in vivo* microenvironment and have the potential to be used as *in vivo* drug delivery vehicle. CeO_2_ is known to show a pH-dependent surface charge^41, 42^. At low pH, it shows a positive surface charge (zeta potential) due to the adsorption of H^+^ ions on its surface, whereas at higher pH, it shows a negative surface charge due to the adsorption of OH^−^ ions on its surface. Given that the positively charged DOX was loaded onto the negatively charged CeO_2_ nanoparticles at physiological pH, the electrostatic interaction between CeO_2_ and DOX was weakened in a low pH environment, and hydrophilic DOX was therefore released from the nanoparticle surface.

The GSH-triggered DOX release can be explained by the GSH-induced reduction of Ce^4+^ to Ce^3+^ and formation of a stable disulphide bridge/Ce(III) complex^43^, leading to DOX release from the nanoparticle surface. Therefore, the highly pH/reduction-responsive release behaviours could be ascribed to the weak electrostatic interaction between CeO_2_ and DOX in a low pH environment, as well as the enhanced drug diffusion caused by stable Ce^3+^/GSSG complex formation. This pH/reduction dual responsiveness is a very useful biological stimulus exploited for triggered drug release, because pH values and intracellular GSH concentrations vary among different cellular organelles. For example, the pH in the endosomes and lysosomes is lower (<5.5) than that in the blood (pH 7.4). By contrast, the intracellular GSH concentration is low in the bloodstream, but is very high inside lysosomes. Therefore, this pH/reduction dual-sensitive drug carrier would not only reduce the amount of drug loss to the blood circulation but would also undergo drug release during the endocytosis process, thereby improving the overall therapeutic efficacy.

The fluorescence microscopy and flow cytometry analyses showed that the CeO_2_/DOX nanoparticles had a higher level of cancer cell uptake compared with free DOX. However, the DOX fluorescence of CeO_2_/DOX might be derived from both the free DOX and the nanoparticle-bound DOX. Free DOX and DOX-conjugated nanoparticles show different cellular uptake mechanisms^44^. Free DOX is known to be transported into cells via a passive diffusion mechanism and can quickly diffuse through the cell membrane, whereas DOX-conjugated nanoparticles are taken up via the endocytosis pathway. In the case of CeO_2_/DOX, DOX fluorescence was observed in both the cytoplasm and nucleus, indicating that the CeO_2_/DOX complexes were initially located within the intracellular compartments (endosomes and lysosomes), and were then released into the cytosol to ultimately enter the nucleus. We further investigated the cellular uptake pathways of CeO_2_/DOX nanoparticles by pre-treating cells with selective endocytosis inhibitors, and found that the nanocomplexes were internalized through caveolin-mediated endocytosis and macropinocytosis.

We have also checked the intracellular DOX release from the CeO_2_/DOX nanoparticles in A2780 cells. Although the intracellular DOX concentration was higher for the free DOX treatment groups after 3 h of treatment, but quickly decayed to 25% of the original concentration at the 24-h mark. Yu *et al*.^45^ also demonstrated a similar pattern of DOX decay (catabolism) in MBT-2 bladder cancer cells within 24 h. By contrast, the CeO_2_/DOX groups showed sustained release of DOX over time and maintained a high intracellular DOX concentration up to 72 h. This result is consistent with the *in vitro* DOX release profile of CeO_2_/DOX. Although the cellular uptake of CeO_2_/DOX was higher than that of the free DOX treatment groups (Fig. 2a,b), the released free DOX concentration was lower (Fig. 2d) after the first 3 h of incubation. However, we found that CeO_2_/DOX showed 6-, 8-, and 9-fold higher DOX retention rates compared with the free DOX treatment groups at 24 h, 48 h, and 72 h, respectively, which could be attributed to the efficient cellular uptake and prolonged drug release behaviour of the complex over time.

Epithelial ovarian cancer which is the cause of most ovarian cancer-related deaths consists of five distinct subtypes of ovarian carcinomas, namely high-grade serous carcinoma (HGSC), clear cell carcinoma (CCC), endometrioid carcinoma (EC), mucinous carcinoma (MC) and low-grade serous carcinoma (LGSC)^46^. High-grade serous carcinoma is the most common type of ovarian carcinomas. Almost half of ovarian CCC and EC are present at stage I and diagnosed. On the other hand, LGSC and MC is the least common among the major types of ovarian carcinomas. Therefore, we have used three different human ovarian cancer cell lines, A2780 (EC model)^47^, SKOV-3 (COC model)^48^, and CAOV-3 (HGSC model)^49^ to check the *in vitro* anti-tumor activity of CeO_2_/DOX nanoparticles. The *in vitro* cytotoxicity and cell apoptosis experiments confirmed that CeO_2_/DOX exhibited higher tumour cell growth inhibition over free DOX in all of the tested human ovarian cancer cells. This confirmed that the released drug was active. CeO_2_ did not induce any significant cell proliferation inhibition or apoptosis, which is consistent with previous studies^20, 21^ using non-targeted nanoceria. However, folic acid-conjugated nanoceria (FA-CeO_2_) could inhibit cell proliferation and induce apoptosis in ovarian cancer cells because of increased cellular internalization^20, 21^. Hijaz *et al*.^21^ also showed that FA-CeO_2_ was more effective in inhibiting tumour growth than CeO_2_ alone in a xenograft mouse model. Moreover, they also showed that the combination of FA-CeO_2_ with cisplatin decreased the tumour burden significantly, even compared to the cisplatin alone-treated group. Therefore, specific targeting of nanoceria and combination with standard chemotherapy holds great potential as an effective therapeutic strategy in ovarian cancer. However, we can expect a better result of the combination therapy in a synergistic or combined mannar in *in vivo* model, because of the antiangiogenic effects of nanoceria^10, 11, 22^. The *in vivo* tumor micro-environment is different from *in vitro* cell culture system and tumor-associated blood vessel formation has been implicated as a key part in the process of growth, invasion, and metastasis of malignancies^50^. Because of the antioxidative property of nanoceria towards normal cells, this CeO_2_/DOX system may offer a novel strategy in cancer treatment by lowering the side effects of doxorubicin, thereby improving the therapeutic outcome^23^. Besides, the CeO_2_/DOX system is stable in *in vivo* microenvironment. Therefore, DOX-loaded nanoceria has the potential to be used as *in vivo* drug delivery vehicle and can be considered as a promising therapeutic agent for cancer treatment.

## Methods

### Materials

Ammonium cerium (IV) nitrate, urea, doxorubicin hydrochloride, and foetal bovine serum (FBS) were purchased from Sigma–Aldrich (St. Louis, MO, USA). Penicillin-streptomycin solution, trypsin-ethylenediaminetetraacetic acid solution, Dulbecco’s modified Eagle medium (DMEM), RPMI, and 1% antibiotic-antimycotic solution were obtained from Life Technologies GIBCO (Grand Island, NY, USA). The bicinchoninic acid protein assay system was obtained from Thermo Scientific (Rockford, IL, USA). Antibodies against caspase 3 (Cell Signaling Technology, Beverly, MA, USA), BCL-2 (Santa Cruz Biotechnology Inc., Santa Cruz, CA, USA), cleaved caspase 9, BAX, and beta-actin (Abcam, Cambridge, MA, USA) were used for immunoblotting.

### Preparation of nanoceria (CeO_2_) and the nanoceria-doxorubicin complex (CeO_2_/DOX)

The protocol for nanoceria (CeO_2_) preparation is described in detail in our previous report^17^. A stock solution of nanoceria was prepared by dissolving an appropriate amount of nanoceria in phosphate-buffered saline (PBS, pH 7.4), followed by sonication; the solution was kept at room temperature. The synthesized nanoceria was characterized by energy-dispersive X-ray spectroscopy (EDS; Oxford EDS-6636), Fourier-transform infrared (FTIR) spectroscopy (Perkin Elmer Spectroscopy GX, PerkinElmer Inc., Waltham, MA, USA), XRD (HRXRD, Brucker D8 Discover) and XPS (Sigma Probe).

For the preparation of CeO_2_/DOX complexes, a DOX solution was prepared by dissolving an appropriate amount of DOX in PBS (pH 7.4), which was then combined with the nanoceria suspension (pH 7.4) and the mixture was stirred overnight in the dark. The mixture solution was subsequently centrifuged at 14,000 *g* for 10 min to remove the excess DOX, and the red-coloured slurry was washed three times with water. The slurry was resuspended in PBS and kept at 4 °C. The synthesized CeO_2_/DOX complexes were characterized by FTIR spectroscopy (Perkin Elmer Spectroscopy GX). The drug-loading efficiency (DLE) and drug-loading content (DLC) were determined by ultraviolet (UV) absorption at 480 nm.

### Transmission electron microscopy (TEM), UV spectroscopy, dynamic light scattering (DLS), and zeta-potential measurements

The primary sizes of CeO_2_ and CeO_2_/DOX complexes were measured by TEM, using a JEM-1200EX microscope at an accelerating voltage of 300 kV. The UV-visible spectra of CeO_2_ and the CeO_2_/DOX complexes were acquired using an Optizen POP (Mecasys, South Korea) instrument. The hydrodynamic sizes and zeta potentials of CeO_2_ and the CeO_2_/DOX complexes were measured in water using a Zetasizer Nano ZS90 (Malvern Instruments, Ltd., Malvern, UK) instrument.

### Cell culture

Human ovarian cancer cells (A2780, SKOV3) were cultured in RPMI supplemented with 10% FBS and 100 U/mL penicillin-streptomycin. CAOV3 human ovarian cancer cells were cultured in DMEM. The cells were cultured in a humidified incubator maintained at 37 °C in the presence of 5% CO_2_.

### In vitro DOX release from CeO_2_/DOX complexes

The *in vitro* release profiles of DOX from CeO_2_/DOX were investigated in neutral and acidic PBS solutions (pH 7.4 and 5.0) with and without glutathionine (GSH, 10 mM), using a dialysis diffusion technique. For the drug release evaluation, an appropriate amount of CeO_2_/DOX (equivalent DOX concentration of 5 μg/mL) was resuspended in 10 mL PBS, sealed in dialysis bags (MWCO 3500 Da), and incubated in PBS (40 mL) at 37 °C with shaking at 100 rpm. At a predetermined time, 4 mL of the incubated solution was taken out and replaced with fresh PBS. The released amount of DOX was determined by measuring the emission fluorescence intensity at 590 nm with an excitation wavelength of 490 nm, using a Gemini EM microplate reader (SpectraMAX, Molecular Devices, Sunnyvale, CA, USA).

### Cellular uptake of CeO_2_/DOX complexes

The cellular uptake experiments were performed using fluorescence microscopy and flow cytometry. For microscopic analysis, A2780 cells were seeded onto glass coverslips placed in six-well plates at 1 × 10^5^ cells per well and incubated overnight at 37 °C in a 5% CO_2_ incubator. The cells were then treated with free DOX or CeO_2_/DOX (DOX concentration = 2 μg/mL) for 3 h. The cells were washed three times with PBS and fixed with fresh 4% paraformaldehyde for 30 min at room temperature. The cell nuclei were stained with DAPI. Coverslips were placed onto glass microscope slides, and DOX uptake was visualized using a fluorescence microscope. To evaluate the cellular uptake pathways, the cells were pre-treated with several endocytosis inhibitors such as 1 mM methyl-β-cyclodextrin (MBCD) or 5 μg/mL chlorpromazine (CPZ), or with a macropinocytosis inhibitor (65 μM LY294002; Sigma–Aldrich) for 30 min. The cells were then treated with the CeO_2_/DOX complexes, as described above, in the presence of the added inhibitors, and DOX uptake was visualized using a fluorescence microscope.

The DOX uptake was also quantified by flow cytometry using a FACS Calibur system, and the data were analysed with Cell Quest software.

### Intracellular DOX release and retention

A2780 cells were exposed to either free DOX or CeO_2_/DOX at an equivalent DOX concentration of 2 μg/mL for 3 h (taken as the 0 time-point). The cells were then washed three times with PBS and further cultured in DOX-free media for 72 h. At various time points, the cells were washed and then lysed for 10 min in lysis buffer and centrifuged at 4000 rpm. The supernatants were collected, and the fluorescence of the released DOX was measured using the Gemini EM microplate reader at 490-nm excitation and 590-nm emission wavelengths.

### Cytotoxicity assay

Cells were seeded (1.5 × 10^4^ cells/well) into 96-well, flat-bottom culture plates and incubated for 24 h at 37 °C in a 5% CO_2_ incubator. The cells were then treated with different concentrations of CeO_2_ (5–100 μM), DOX (0.25–5 μg/mL), or CeO_2_/DOX (equivalent DOX concentration of 0.25–5 μg/mL) for 3 h in a humidified incubator at 37 °C in the presence of 5% CO_2_. The medium was then replaced with fresh medium containing 10% FBS, and the cells were further cultured without DOX and nanoparticles for 72 h. A cell viability assay was performed using Cell Counting Kit-8 (CCK-8, Dojindo Laboratories, Kumamoto, Japan), and the absorbance was read at a wavelength of 450 nm using a microtitre plate reader (Multiskan FC, Thermo Fisher Scientific Inc., Waltham, MA, USA).

### Acridine orange/ethidium bromide (AO/EB) staining

DOX-induced apoptosis was detected by AO/EB double staining. Cells were seeded in 24-well plates and incubated overnight at 37 °C in a 5% CO_2_ incubator. The cells were then treated with either free DOX (0.25 μg/mL) or CeO_2_/DOX (equivalent DOX concentration of 0.25 μg/mL) for 3 h at 37 °C. The cells were washed with PBS and further cultured for 72 h with fresh medium containing 10% FBS without DOX. Finally, the cells were washed with PBS and stained with the AO/EB mixture (100 μg/mL) for 5 min, followed by another three washes with PBS. The cells were visualised using an inverted fluorescence microscope under an excitation wavelength of 490 nm and an emission wavelength of 530 nm for AO staining, and an excitation wavelength of 520 nm and emission wavelength of 590 nm for EB staining.

### Assessment of apoptotic cell populations

Cells were seeded (1.5 × 10^4^ cells/well) into 96-well, flat-bottom culture plates and incubated for 24 h at 37 °C in a 5% CO_2_ incubator. The cells were then treated with DOX (0.25 μg/mL), or CeO_2_/DOX (equivalent DOX concentration of 0.25 μg/mL) for 3 h in a humidified incubator at 37 °C in the presence of 5% CO_2_. The medium was then replaced with fresh medium containing 10% FBS, and the cells were further cultured without DOX and nanoparticles for 72 h. Cell death was analyzed using FITC conjugated Annexin V and propidium iodide with an Apoptosis detection kit (Komabioteh, Seoul, South Korea) according to the manufacturer’s instructions. Cells were characterized using cytoFLEX flow cytometer.

### Immunoblotting

The cells were lysed in radioimmunoprecipitation lysis buffer containing protease and phosphatase inhibitors. Equal amounts of protein were resolved by 12–13% sodium dodecyl sulphate-polyacrylamide gel electrophoresis, and the proteins were electrophoretically transferred to polyvinylidene fluoride membranes. The membranes were blocked at room temperature with 6% non-fat dry milk for 2 h to prevent non-specific binding, and then incubated with primary antibodies overnight at 4 °C. Immunoreactivity was detected through sequential incubation with horseradish peroxidase-conjugated secondary antibodies and enhanced chemiluminescence reagents.

### Statistical analysis

All experiments were performed at least in triplicate, and statistical analyses were performed using one-way analysis of variance followed by Student *t*-tests. The level of significance was set at P < 0.05.

## Electronic supplementary material

Supplementary Information
