# Supplementary material for: Nanoceria-mediated delivery of doxorubicin enhances the anti-tumour efficiency in ovarian cancer cells via apoptosis
Source: Sci Rep. 2017 Aug 25;7:9513. doi: 10.1038/s41598-017-09876-w (PMC5572723; doi:10.1038/s41598-017-09876-w)

**Nanoceria-mediated delivery of doxorubicin enhances the anti-tumour efficiency in ovarian cancer cells via apoptosis**

Joydeep Das, Yun-Jung Choi, Jae Woong Han, Abu Musa Md Talimur Reza and Jin-Hoi Kim*

Dept. of Stem Cell and Regenerative Biotechnology, Humanized Pig Research Center (SRC), Konkuk University, Seoul 143-701, South Korea

**Running title:** Nanoceria mediated drug delivery

**Corresponding author:**

**Jin-Hoi Kim*:** [jhkim541@konkuk.ac.kr](mailto:jhkim541@konkuk.ac.kr); Fax: +82-2-458-5414

Department of Animal Biotechnology,

Humanized Pig Research Center (SRC),

Konkuk University, Seoul 143-701, South Korea

**Supplementary Figure legends**

**Fig. s1:** (a) XRD pattern of nanoceria. (b) High-resolution XPS spectrum of nanoceria. (c) DOX release profiles of the CeO_2_/DOX nanoparticles in PBS with 10 % FBS at 37°C. All values are expressed as mean ± SD.

**Fig. s2.** Hydrodynamic size, polydispersity index (PDI) and zeta potential of CeO_2_ and CeO_2_/DOX nanoparticles. All values are expressed as mean ± SD.

**Fig. s3.** Cellular uptake mechanism of CeO_2_/DOX in A2780 cells. Cells were either cultured at 37 °C as a negative control (Cont) or pretreated with MBCD, chlorpromazine, or LY294002 for 30 mints. After that cells were treated with 2 μg/mL equivalent doxorubicin concentration for 3 hrs in presence of the endocytosis inhibitors and the CeO_2_/DOX internalization was measured by fluorescence microscopy.

**Fig. s4:** Acridine orange/ethidium bromide (AO/EB) staining after incubation of the cells with a 0.25 μg/mL equivalent DOX concentration for 3 h, followed by washing and further culture for 72 h in DOX-free medium. (a) A2780 cells; (b) CAOV3 cells and (c) SKOV3 cells.

**Fig. s5:** Full length gel images for the Western blot analysis with A2780 cell lysates.

**Fig. s6:** Full length gel images for the Western blot analysis with CAOV3 cell lysates.

**Fig. s7:** Full length gel images for the Western blot analysis with SKOV3 cell lysates.


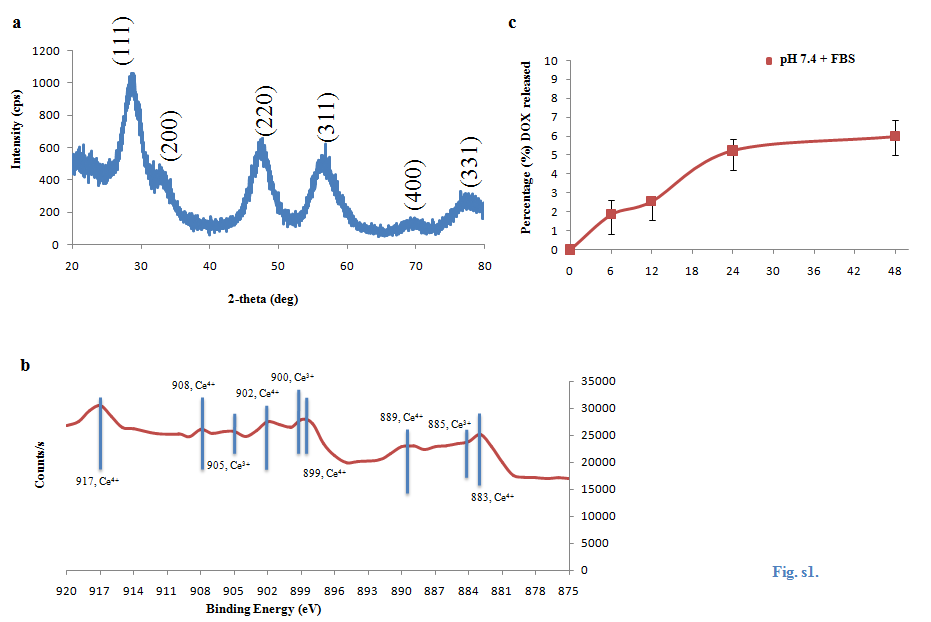


**
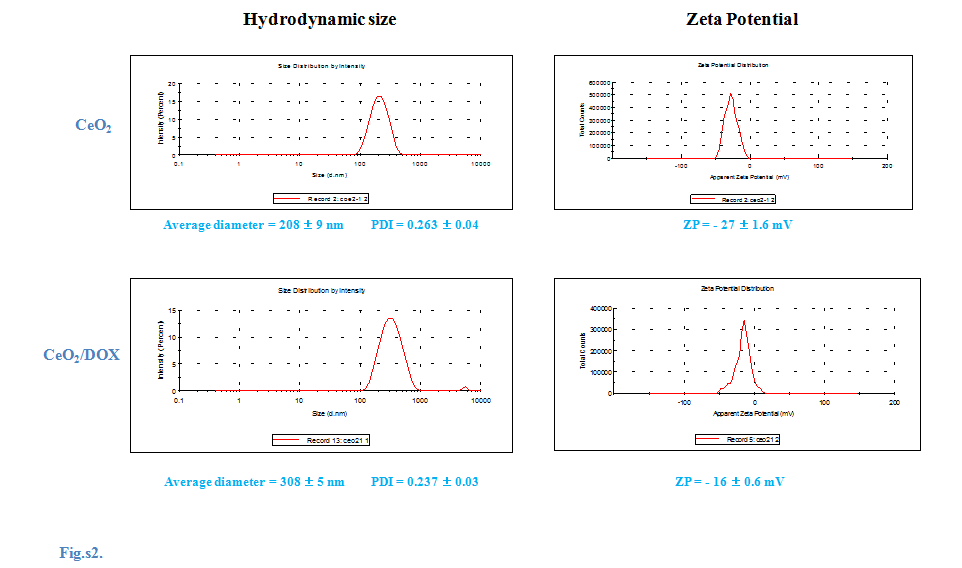
**

**
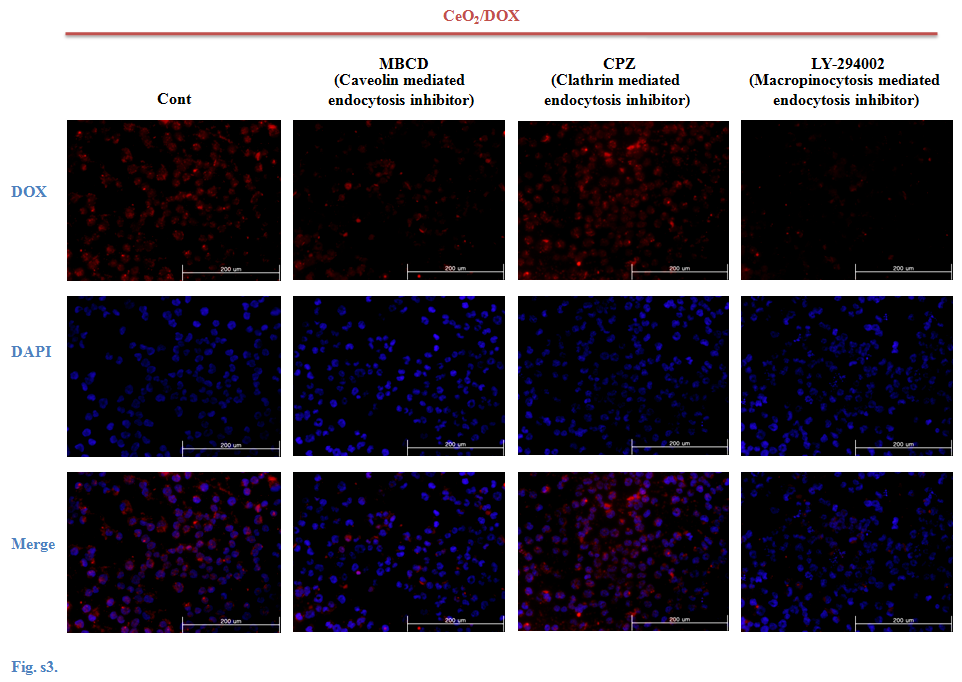
**

**
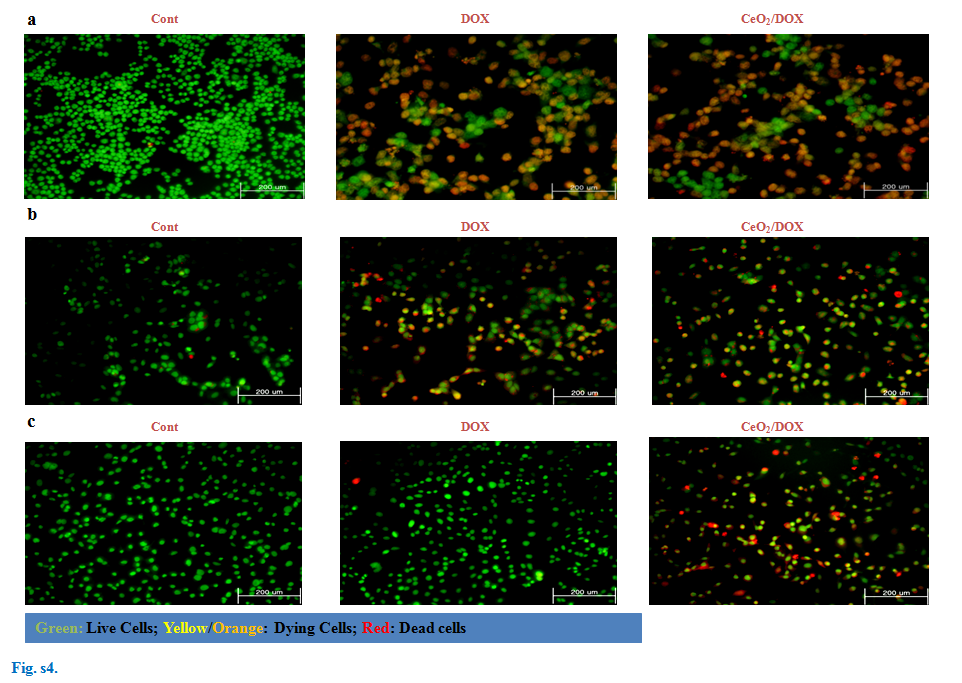
**


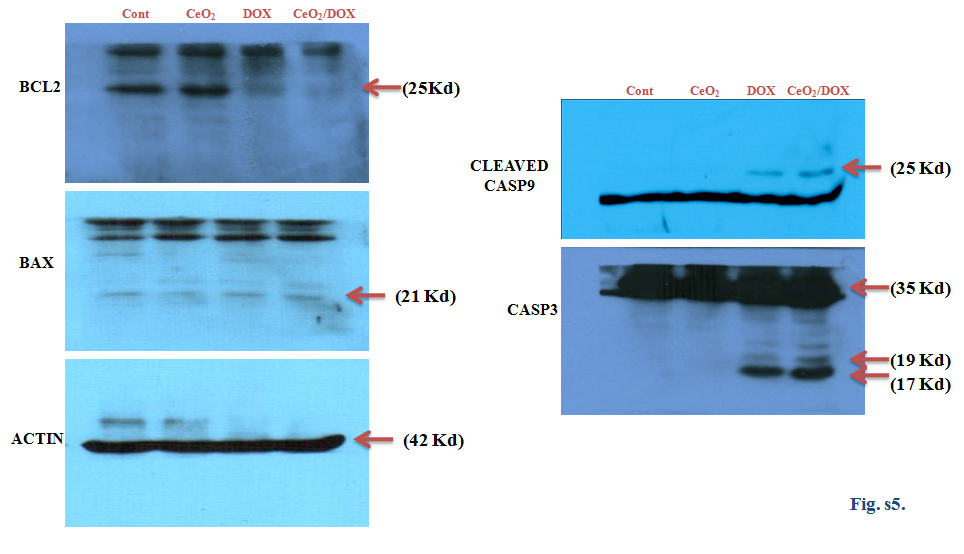


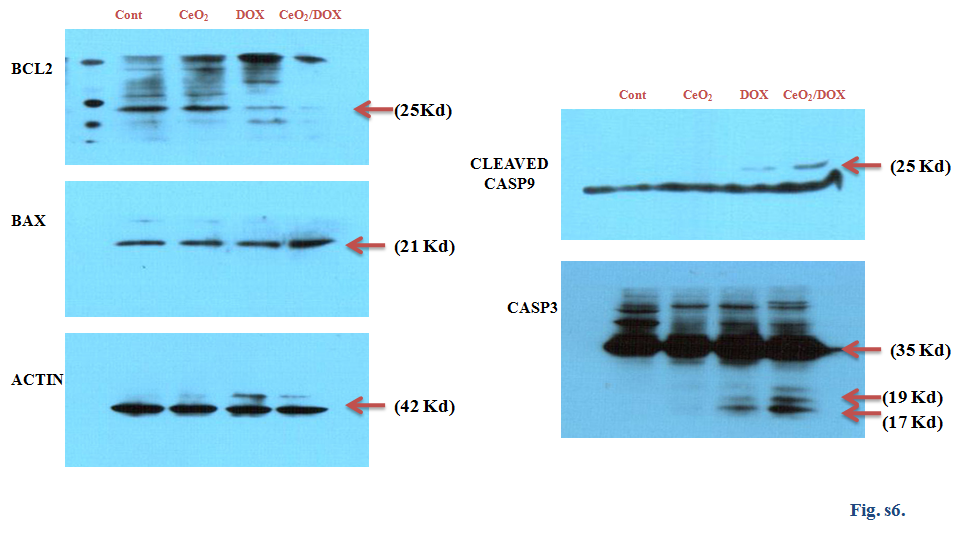


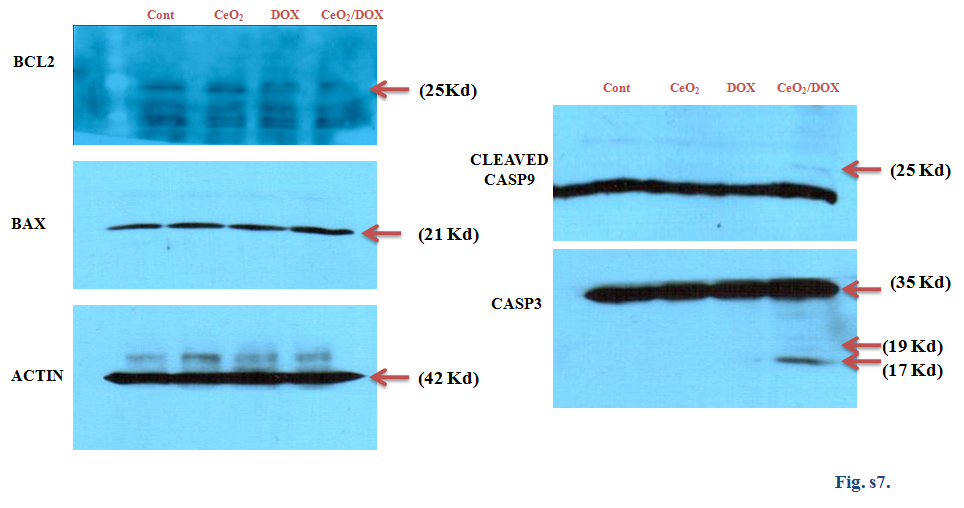

Supplement: Supplementary file 1 — Supplementary Information [file 41598_2017_9876_MOESM1_ESM.doc]
